# Tertiary Lymphoid Organs in Takayasu Arteritis

**DOI:** 10.3389/fimmu.2016.00158

**Published:** 2016-04-22

**Authors:** Marc Clement, Adrien Galy, Patrick Bruneval, Marion Morvan, Fabien Hyafil, Khadija Benali, Nicoletta Pasi, Lydia Deschamps, Quentin Pellenc, Thomas Papo, Antonino Nicoletti, Karim Sacre

**Affiliations:** ^1^INSERM U1148, Université Paris Diderot, PRES Sorbonne Paris Cité, Paris, France; ^2^Département de Médecine Interne, Hôpital Bichat, Assistance Publique Hôpitaux de Paris, Université Paris Diderot, PRES Sorbonne Paris Cité, Paris, France; ^3^Département de Pathologie, Hôpital Européen Georges Pompidou, Assistance Publique Hôpitaux de Paris, Université Paris Descartes, PRES Sorbonne Paris Cité, Paris, France; ^4^Département de Médecine Nucléaire, Hôpital Bichat, Assistance Publique Hôpitaux de Paris, Université Paris Diderot, PRES Sorbonne Paris Cité, Paris, France; ^5^Département de Radiologie, Hôpital Bichat, Assistance Publique Hôpitaux de Paris, Université Paris Diderot, PRES Sorbonne Paris Cité, Paris, France; ^6^Département de Pathologie, Hôpital Bichat, Assistance Publique Hôpitaux de Paris, Université Paris Diderot, PRES Sorbonne Paris Cité, Paris, France; ^7^Département de Chirurgie Vasculaire, Hôpital Bichat, Assistance Publique Hôpitaux de Paris, Université Paris Diderot, PRES Sorbonne Paris Cité, Paris, France; ^8^INSERM U1149, Laboratoire d’excellence INFLAMEX, Université Paris Diderot, PRES Sorbonne Paris Cité, Paris, France; ^9^Département Hospitalo-Universitaire FIRE (Fibrosis, Inflammation and Remodelling in Renal and Respiratory Diseases), Université Paris Diderot, PRES Sorbonne Paris Cité, Paris, France

**Keywords:** tertiary lymphoid organs, Takayasu arteritis, B cells, immunopathogenesis

## Abstract

**Objective:**

The role of B cells in the pathogenesis of Takayasu arteritis (TA) is controversial. We aimed to study the presence of tertiary lymphoid organs (TLOs) in the aortic wall of TA patients.

**Methods:**

Hematoxylin and eosin-stained sections from aorta specimens from patients with TA were screened for TLOs. The presence of B cell aggregates (CD20), follicular dendritic cells (FDCs, CD21), and high endothelial venules (HEVs, PNAd) was investigated by immunohistochemistry. Immune cells from the adventitial layer of one patient were characterized by flow cytometry. Demographic, medical history, laboratory, imaging, treatment, and follow-up data were extracted from medical records.

**Results:**

Aorta specimens from Bentall procedures were available from seven patients (5 females, aged 22–57 years) with TA. Surgical treatment was performed at TA diagnosis (*n* = 4) or at a median of 108 months (84–156) after TA diagnosis. Disease was active at surgery in four patients according to NIH score. B cell aggregates-TLOs containing HEVs were observed in the adventitia of all but one patient. Of note, ectopic follicles containing CD21^+^ FDCs were found in all patients (4/4) with increased aortic ^18^F-fluoro-deoxyglucose (FDG) uptake before surgery but were absent in all but one patients (2/3) with no FDG uptake. In addition, flow cytometry analysis confirmed the accumulation of memory/germinal center-like B cells in the adventitial layer and showed the presence of antigen-experienced T follicular helper cells.

**Conclusion:**

Ectopic lymphoid neogenesis displaying functional features can be found in the aortic wall of a subset of patients with active TA. The function of these local B cell clusters on the pathogenesis of TA remains to be elucidated.

## Introduction

Takayasu arteritis (TA) is a rare primary vasculitis affecting large arteries, especially the aorta, the aortic arch, and its main branches. The etiology of TA is unknown even if it has been proposed that infectious agents play a significant role in the pathogenesis of this disease (1). In addition, while T cells exhibiting a Th1 profile have been implicated in the pathogenesis of TA (1, 2), the role of humoral immunity remains to be elucidated.

Tertiary lymphoid organs (TLOs) are ectopic lymphoid structures that form at sites of chronic inflammation through a process referred as lymphoid neogenesis. TLOs have morphologic features of secondary lymphoid organs including post-capillary high endothelial venules (HEVs) allowing homing of naive cells in the T cell area, an interface between T and B cell zones and germinal center (GC) areas. TLOs have been described in autoimmunity, microbial infection, cancer, chronic allograft rejection, atherosclerosis models, and abdominal aortic aneurysms (3–5). Studies have pinpointed the presence of lymphoid aggregates in the aortic wall of TA patients without distinguishing them from granulomas and have suggested their implication in the physiopathology of TA (6, 7). In addition, pathogenic B cells producing autoantibodies against endothelial cells are found in the blood stream of active TA patients, which suggests that these B cells could be activated within the adventitial layer before recirculating (8). Finally, some TA patients with active disease have a dramatic increase of circulating plasmablasts that are efficiently targeted by depleting anti-CD20 antibodies (9).

We aimed at characterizing the peri-aortic lymphoid aggregates developing in TA patients and at analyzing whether their presence could be associated with the activity of the disease.

## Materials and Methods

### Patients

We conducted a retrospective multicenter study of patients with TA in whom aortic surgery was performed and aortic tissue specimen was available for analysis. All patients fulfilled the American College of Rheumatology and the Sharma-modified Ishikawa criteria for TA (10, 11). Demographic, medical history, laboratory, imaging findings, including ^18^F-fluoro-deoxyglucose positron emission tomography (FDG-PET), computed tomography angiography (CTA), and/or magnetic resonance imaging (MRI), treatment, and follow-up data were extracted from medical records. The patients’ clinical, laboratory, and imaging data, as well as treatments were analyzed at disease onset and at time of surgery. Routine laboratory tests, including C-reactive protein levels, were collected, and disease activity was defined according to the NIH criteria (12). Disease was considered active if NIH score was 2 or more, and inactive otherwise.

### Immunohistochemistry on Aortic Samples

Aorta tissues were fixed in 4% PFA, embedded in paraffin, and sectioned at 6 μm. The sections were deparaffinized in toluene, hydrated in ethanol, and incubated in retrieval reagent (R&D Systems) for 20 min. After blocking in 5% BSA, the slides were incubated with purified primary antibody (mouse anti-human CD20, clone L-26; rabbit anti-human CD3, polyclonal, Dako; rat anti-human PNAd, MECA-79, BD Biosciences; rabbit anti-human CD14, clone EPR3653; rabbit anti-human CD21, clone EP3093, Abcam; mouse anti-human CD15, clone HI98, Biolegend) overnight at 4°C. After several washes with PBS, sections were incubated with the appropriate secondary antibody (polyclonal goat anti-rabbit DyLight649; goat anti-mouse rhodamine; goat anti-rat rhodamine; donkey anti-mouse rhodamine, Jackson Immunoresearch) at RT for 30 min and then cover mounted using Prolong Gold Antifade Reagent^®^ (Invitrogen) for microscopy. The resulting fluorescence was detected with a Zeiss Axiovert 200 M microscope equipped with an AxioCam MRm version 3 camera, an ApoTome^®^ system, and AxioVision^®^ image capture software.

### Flow Cytometry Analysis

Adventitial layer samples from the aorta were weighed, cut into small pieces (<1 mm), and digested using a previously described protocol (13). After a wash step, the cells were incubated with LIVE/DEAD^®^ Fixable Yellow Dead Cell Stain Kit (Molecular Probes) and stained with the following antibodies: mouse anti-human CD19 brilliant violet 785, clone HIB19 (Biolegend); mouse anti-human CD45 eFluor 605, clone HI30 (eBioscience); mouse anti-human CD3 eFluor 450, clone OKT3 (eBioscience); mouse anti-human CD4 PE-CF594, clone UCHL1 (BD Biosciences); mouse anti-human HLA-DR APC-H7, clone L234; mouse anti-human CD27 brilliant violet 421, clone M-T271; mouse anti-human IgD APC, clone IA6-2; mouse anti-human CD95 FITC, clone DX2 (BD Biosciences); rate anti-human CXCR5 FITC, clone rf8b2 (BD Biosciences); mouse anti-PD-1 PerCP-Cy 5.5, clone EH1 2.1 (BD Biosciences); mouse anti-human CD45RA V450, clone HI100 (BD Biosciences); mouse anti-human Bcl6 PE, clone IG191/A8 (Biolegend). Flow cytometry analysis was performed using an LSR II flow cytometer (BD Biosciences). Data were analyzed with DIVA (BD Biosciences) and FlowJo (TreeStar) software.

### Ethical Statement

Our study is a retrospective human non interventional study where subjects were not assigned to treatment; they were assigned to a diagnosis strategy within the current practice; the study involved products with a marketing authorization that are prescribed in the usual manner and used in accordance with authorizations by French agencies; epidemiological methods were used to analyze the data; and information used in the study were collected for clinical care. According to the Public Health French Law (art L 1121-1-1, art L 1121-1-2), written consents and IRB approval are not required for human non interventional studies. Patients were however informed that data collected in medical records might be used for research study in accordance to privacy rule. The study protocol conforms to the ethical guidelines of the 1975 Declaration of Helsinki.

## Results

### Patients’ Characteristics at Disease Onset and Medical Treatment

Aorta specimens were available from 16 patients with TA who underwent surgery between 2009 and 2014. Nine patients were excluded because diagnosis criteria for TA were not met (*n* = 7) or data were missing (*n* = 2). Seven patients (five females and two males) who fulfilled American College of Rheumatology and Ishikawa criteria for TA were studied. The median age at TA diagnosis was 34 years (22–57). Diabetes mellitus and dyslipidemia were present in cases 1 (14.3%) and 2 (28.6%), respectively. Tobacco use was reported in four (57.1%) patients. No patients had past history of tuberculosis. The median body mass index was 23.5 kg/m^2^ (21.1–33.3). The country of origin was France (*n* = 4), Morocco (*n* = 1), Mali (*n* = 1), and West Congo (*n* = 1).

At disease onset, patients suffered from fatigue (*n* = 4), headache (*n* = 2), carotidodynia (*n* = 2), weight loss (*n* = 1), dizziness (*n* = 1), arthritis (*n* = 1), visual disturbance (*n* = 1), and erythema nodosum (*n* = 1). No patients had fever. One patient was asymptomatic but had asymmetric blood pressure. Hypertension was present at onset (*n* = 4) or occurred (*n* = 1) during the course of the disease. The median delay between the onset of first symptoms and the diagnosis of disease was 29 months (3–96).

Serum C-reactive protein levels were raised in five (71.4%) patients [median 68 mg/L (11–106)]. At TA diagnosis, disease was considered active (NIH score ≥2) in all patients. CT angiography of the aorta was performed at TA diagnosis and showed multiple arterial lesions in all patients. Aorta was involved in all cases (Table 1).

**Table 1:** **Patients’ baseline characteristics**.

Patients	P01	P02	P03	P04	P05	P06	P07
Sex	F	F	M	F	M	F	F
Age	57	26	22	41	42	34	32
Smoker	Yes	Yes	No	No	Yes	No	Yes
Dyslipidemia	Yes	No	No	No	No	Yes	No
Hypertension	Yes	No	Yes	No	Yes	Yes	Yes
Diabetes	No	No	No	Yes	No	No	No
BMI (kg/m^2^)	24	24.8	22.8	23	26.6	33.3	21.1
Diagnosis delay (months)	11	3	29	82	96	32	13
Systemic features	Yes	No	No	Yes	Yes	Yes	Yes
Vascular features	No	Yes	Yes	Yes	No	Yes	Yes
C-reactive protein (mg/L)	68	1	4	48	106	11	75
Aortic lesions (type)	IIa (C^+^P^−^)	V (C^+^P^+^)	V (C^−^P^−^)	V (C^−^P^−^)	IIb (C^−^P^+^)	V (C^−^P^−^)	IIb (C^−^P^−^)
Disease activity score	3	2	2	4	3	4	4

*BMI, body mass index; the type of aortic lesions was defined according to international criteria (14); the disease activity score was assessed according to NIH criteria (12)*.

*Systemic features referred to fatigue (*n* = 4), headache (*n* = 2), weight loss (*n* = 1), dizziness (*n* = 1), arthritis (*n* = 1), visual disturbance (*n* = 1), or erythema nodosum (*n* = 1)*.

*Vascular features referred to carotid or subclavian bruit (*n* = 4), claudication with a diminished or absent pulse (*n* = 4) and carotidodynia (*n* = 2)*.

All patients received glucocorticoids as first-line treatment. Methotrexate (*n* = 3), infliximab (*n* = 2), azathioprine (*n* = 1), and tocilizumab (*n* = 1) were prescribed during follow-up in four patients.

### Patients’ Characteristics at Surgery

Surgery was performed at the time of first diagnosis in four patients or during follow-up (84–156 months) in three patients. Disease was active at time of surgery in four patients with a NIH score ranging from 2 to 3, including recent ischemic vascular claudication (*n* = 2), new upper limb bruit (*n* = 1), and/or C-reactive protein >10 mg/L (*n* = 3). Aortic CT angiography displayed an increased circumferential aorta wall thickness (3.5–16 mm, median 7 mm). PET showed an increased FDG uptake in the vascular wall of the thoracic aorta wall in four cases. Three patients, including one with active disease, were still receiving glucocorticoids at time of surgery with a daily dose <10 mg. One patient was under tocilizumab. Antiplatelet treatment and statin were given in cases 3 and 4, respectively (Table 2).

**Table 2:** **Patients’ characteristics at surgery**.

Patients	P01	P02	P03	P04	P05	P06	P07
**General features**							
Sex	F	F	M	F	M	F	F
Age at diagnosis (years)	57	26	22	41	42	34	32
Age at surgery (years)	57	33	22	41	51	34	45
C-reactive protein (mg/L)	35	33	4	9	4	11	1
Disease activity score	2	2	2	0	1	3	0
Corticosteroid treatment	No	Yes	No	No	Yes	No	Yes
Immunosuppressive drugs	No	No	No	No	No	No	Yes
Antiplatelet treatment	No	Yes	No	No	Yes	No	Yes
Statin	No	Yes	Yes	Yes	No	Yes	No
**Aortic disease**							
Wall thickness (mm, CT scan or MRI)	10	NA	7	5	16	5	3.5
Wall contrast enhancement (CT scan or MRI)	NA	NA	No	No	Yes	NA	Yes
PET (SUV max)	4.8	0	1.3	0	5.0	0	7.0
Apparent *in situ* aortic inflammation	Yes	Yes	Yes	No	No	Yes	Yes
**Aorta histological findings**							
Mononuclear cells infiltrate	M, A	M, A	I, M, A	A	M, A	A	I, M, A
Multinuclear giant cells	M	No	M	No	No	No	M
Sclerosis	A	A	I, M, A	M, A	I, A	I, A	A
Neovascularization	No	M	M	M, A	M, A	No	No
Plasma cell	No	M, A	No	No	M	A	No
Wall thickness	A	A	I	No	I, A	I, A	A
Coexistent atheroma	No	No	No	No	No	Yes	Yes
Vasa vasorum thickening	Yes	No	No	No	No	No	Yes
Interruption of elastic lamina	Yes	Yes	Yes	Yes	Yes	Yes	Yes
Granuloma	M	No	No	No	No	No	M
Tertiary lymphoid organ	A	A	A	No	A	A	A
CD21^+^ FDCs	++	++	+	−	+	−	++

*PET, positron emission tomography; CT, computed tomography; MRI, magnetic resonance imaging; FDCs, follicular dendritic cells; SUV max, maximal standard uptake values; I, intima; M, media; A, adventitia; NA, not available*.

*The disease activity score was assessed according to NIH criteria (12)*.

Fourteen vascular procedures were done in seven patients (1–3 per patient, mean 1.7). Indications for surgery were aortic regurgitation due to thoracic aneurysm (*n* = 7) and abdominal aorta stenosis with lower limb chronic ischemia (*n* = 1).

Specimens from the thoracic aorta were obtained from Bentall procedures in all cases. At surgery, peri-aortic inflammation was obvious in five patients. Microscopically, prominent fibrosis was observed in intimal (*n* = 3), medial (*n* = 2), and adventitial (*n* = 7) layers, associated with increased intimal (*n* = 3) and adventitial (*n* = 5) thickness. Medial or adventitial inflammation was seen in all cases and involved mononuclear cells (*n* = 7), giant cells (*n* = 3), and plasma wall cells (*n* = 3). The deep portion of the media (*n* = 4) and adventitia (*n* = 2) contained neovessels. Atherosclerotic lesions were detected in two aortic specimens (Table 2).

### Tertiary Lymphoid Organs in the Aortic Wall

**Figure 1:**
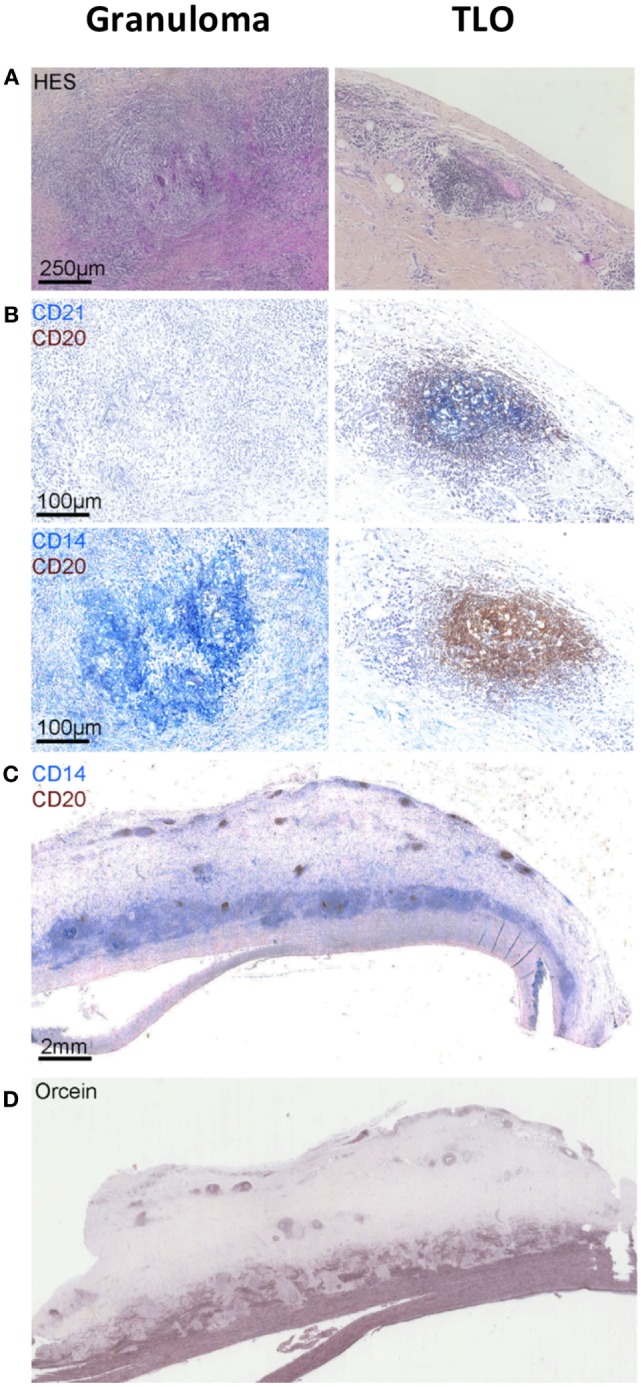
**Granulomas versus tertiary lymphoid organs (TLOs) in TA patients**. Hematoxylin and eosin staining showing the presence of granuloma [first column **(A)**] in the aortic wall identified as CD14^++^CD20^−^CD21^−^ cell aggregates by using a combination of CD21 (blue) CD20 (brown) [first column **(B)**] and CD14 (blue) CD20 (brown) [first column **(B)**] staining. Other cell aggregates also visible on hematoxylin and eosin-stained sections [second column **(A)**] are instead TLOs structures displaying few CD14^+^ cells (blue), many CD20^+^ B cells (brown), and CD21^+^ FDCs (blue) **(B)**. Representative picture **(C)** showing the distribution of granulomas (CD14^++^) in contact with the medial layer and TLOs (CD20^++^) located deeper within the adventitial layer. Orcein staining **(D)** suggests that granulomas are physically implicated in medial destruction, whereas TLOs might develop in response to sustained inflammation in the adventitia.

**Figure 2:**
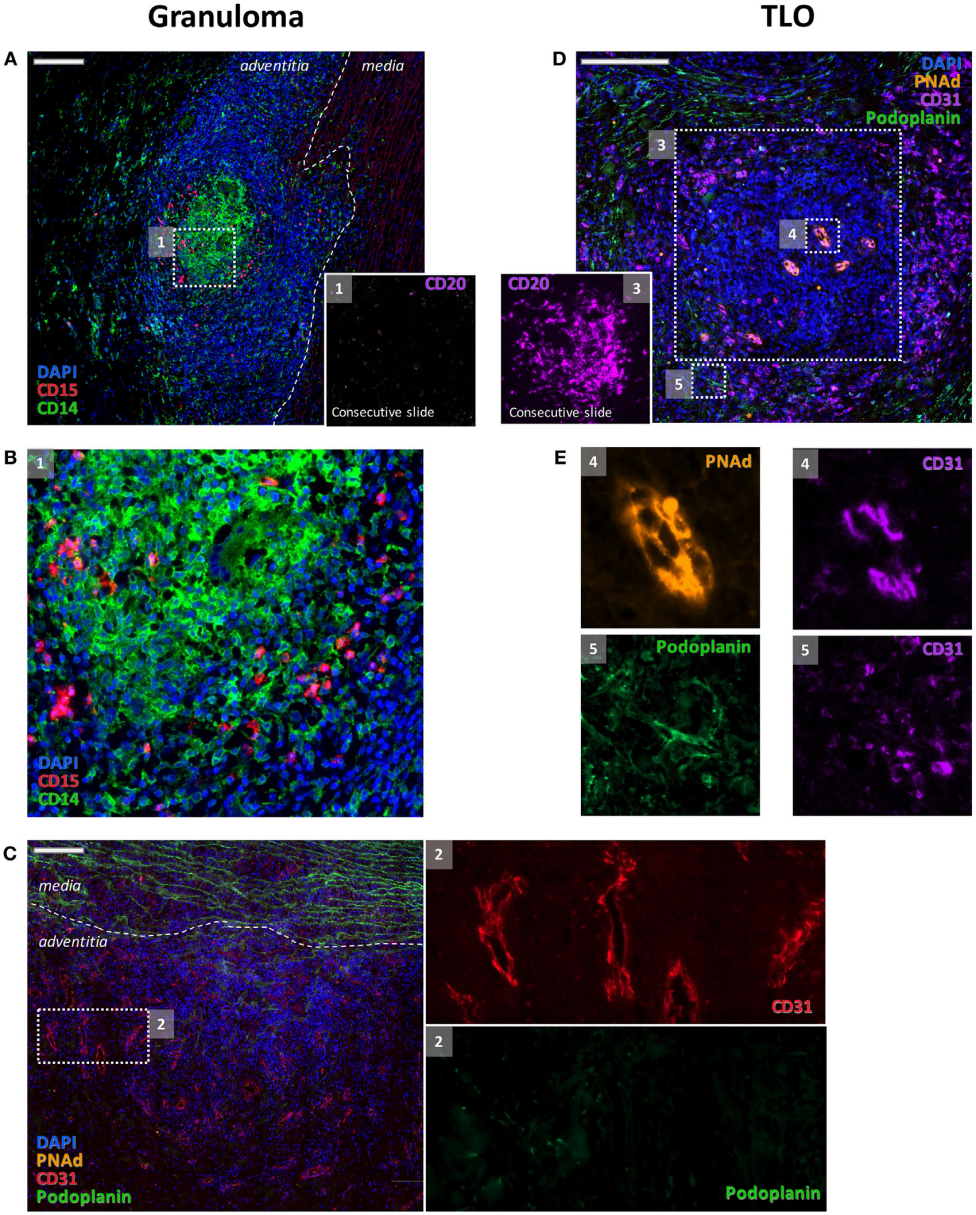
**Distinct features of granulomas and TLOs**. Granulomas were made of clusters of CD14^+^ cells and of CD15^+^ cells **(A,B)** where very few B cells (inset 1) could be detected. Granulomas were also characterized by an intense angiogenesis [CD31^+^ vessels **(C)** and inset 2]. These adventitial neovessels were PNAd^−^ **(C)**. Few podoplanin + lymphatic vessels were also associated with blood vessels [**(C)** and inset 2]. At variance, CD31^+^ blood vessels with endothelial cells displaying a high endothelial venule phenotype and expressing PNAd [**(E)** and inset 4] were systematically observed within the B cell clusters [**(D)** and inset 3] that are composed of TLOs. A rich network of CD31^+^ Podoplanin^+^ lymphatics was detected around TLOs [**(E)** and inset 5].

**Figure 3:**
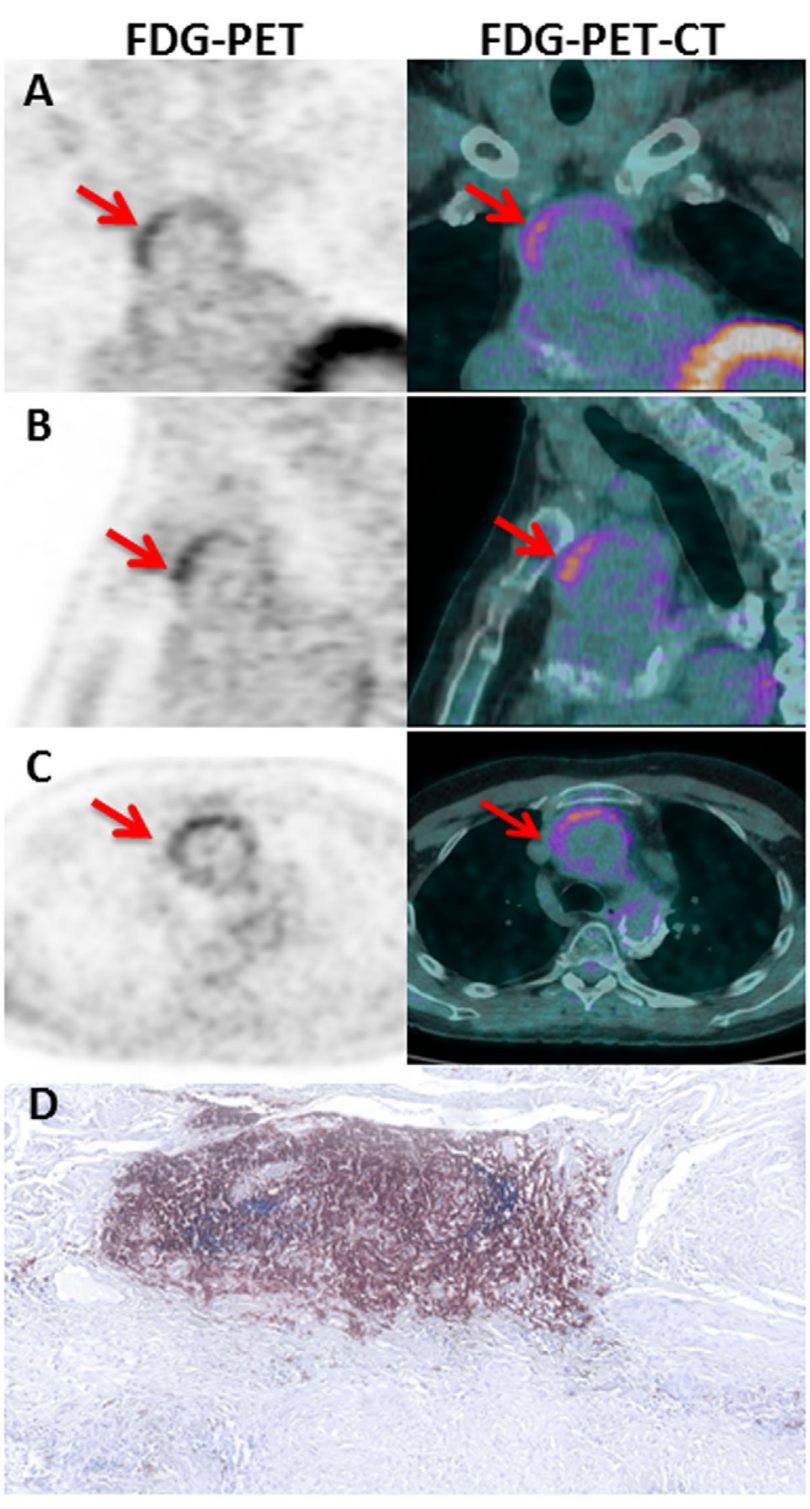
**FDG-PET-CT imaging in TA patient**. Coronal **(A)**, sagittal **(B)**, and axial **(C)** slices of the thoracic aorta with FDG-PET imaging in P05. Note the presence of a linear, high FDG uptake in the ascending thoracic aorta on PET corresponding to the anterior region of the aortic wall from an aortic aneurysm on corresponding CT images. The left and the right imaging are, respectively, ^18^FDG PET and fused PET/CT images showing high tracer uptake on the ascending aorta (Red arrows). Hematoxylin and eosin staining **(D)** of CD21^+^ (blue) CD20^+^ (brown) TLOs in the adventitial layer of the aortic wall of the same P05 patient.

Combination of CD14, CD20, and CD21 staining allowed identification of active TLOs (>90% CD14^low^CD20^+^CD21^+^) and granulomas (>90% CD14^++^CD20^−^CD21^−^) in the vessel wall. TLOs were observed in the aortic wall in all but one patient. Granulomas were made of macrophages/neutrophils (CD14^+^/CD15^+^) and identified in only two patients. Interestingly, distribution patterns of granulomas and TLOs were distinct: granulomas were in contact with the medial layer, whereas TLOs were located deeper in the adventitial layer (Figure 1). Of note, TLOs (CD20^+^ clusters) contained a dense network of HEVs (PNAd^+^) able to recruit naive cells from the periphery. No HEVs were detected within or around CD14^+^/CD15^+^ granulomas (Figure 2). Of note, the presence of TLOs in the aortic wall was associated with FDG uptake measured by PET-CT scanner (Figure 3). Indeed, ectopic follicles containing CD21^+^ follicular dendritic cells (FDCs) were found in all patients with increased aortic FDG uptake before surgery, but were absent in all but one (2/3) patients with no FDG uptake (Table 2).

### Memory B Cells and Antigen-Experienced T Follicular Helper Cells in the Aortic Wall

Patient 1 (P01) had a Bentall procedure for aortic regurgitation due to thoracic aortic aneurysm (Tables 1 and 2, for clinical details). CTA and FDG-PET revealed active aortic lesions in the ascending aorta and the aortic arch at surgery time.

**Figure 4:**
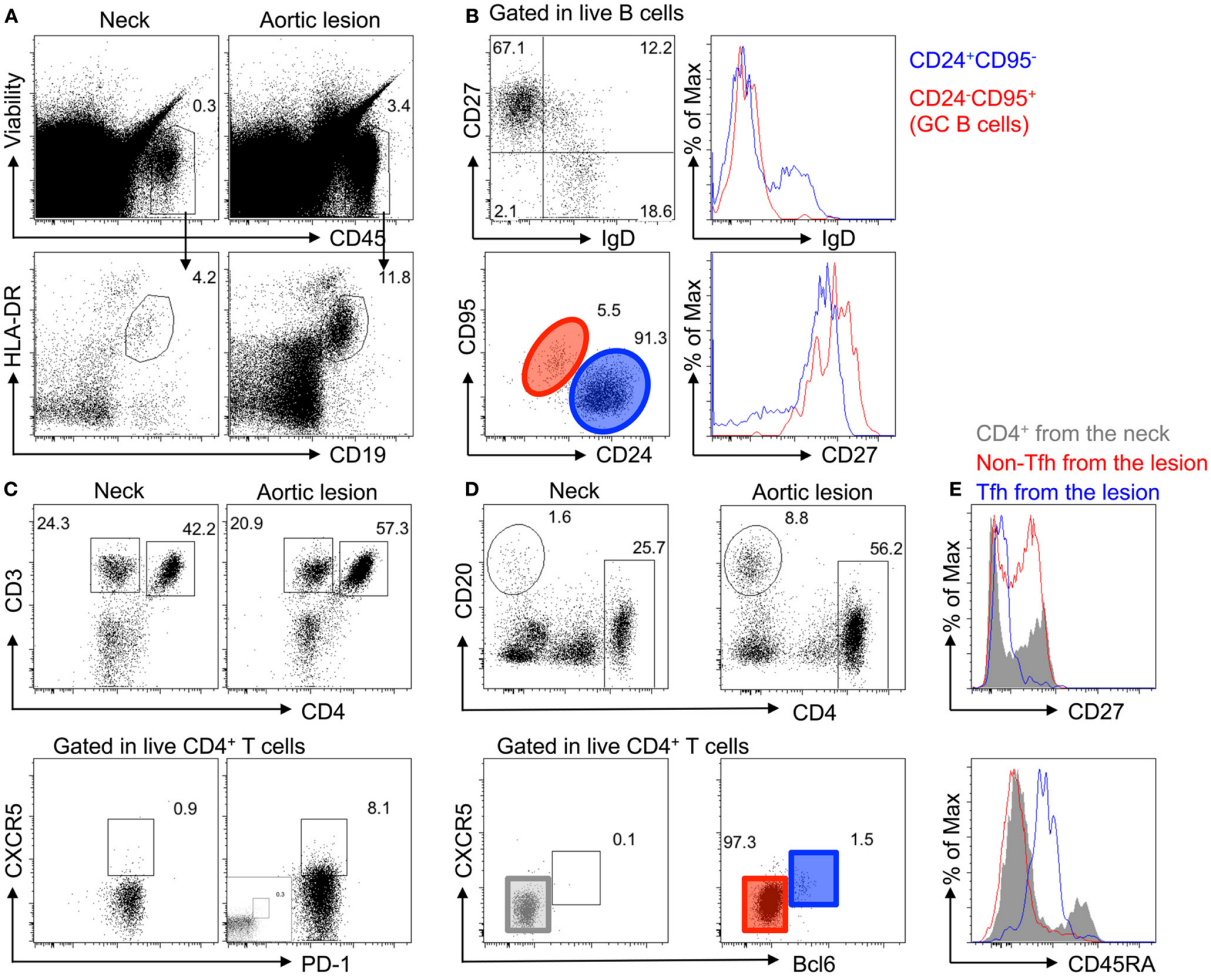
**Accumulation of memory B cells, germinal center B cells, and CD4^+^ T follicular helper cells in the adventitia of TA diseased aortas**. **(A)** Accumulation of immune cells (Viability^−^CD45^+^) and B cells (HLA-DR^+^CD19^+^) analyzed by flow cytometry in the adventitial layer of the core of the aortic lesions of a TA patient (P01) as compared to the neck of the same sample. **(B)** Characterization of B cells in the adventitia of the aortic lesion by flow cytometry shows that most B cells have a memory phenotype (CD27^+^IgD^−^) with some harboring a germinal center phenotype (CD95^+^CD24^−^IgD^−^CD27^high^). **(C)** Flow cytometry analysis of adventitial tissue samples reveals an increase in CD4^+^ T cells in the aortic lesion (top panel), as well as the presence of CD4^+^ T cells harboring a Tfh cell phenotype (PD1^+^CXCR5^+^), as compared to aortic tissues sampled in the aneurysmal neck. **(D)** CXCR5^+^CD4^+^ T cells from the adventitia of the core aortic lesion are Bcl6^high^. **(E)** CD4^+^ T cells from the adventitia of the neck (gray), non-Tfh cells (CXCR5^−^Bcl6^−^; Red), and Tfh cells (CXCR5^+^Bcl6^high^; blue) were analyzed for CD27 and CD45RA expression. As compared to CD4^+^ T cells from the neck, which are a mixture of naive and antigen-experienced (CD27^−^) and memory cells (CD45RA^−^), the adventitia of the core of the aortic lesion contains more antigen-experienced and no naive T cells (CD45RA^+^). Interestingly, Tfh cells in the adventitia are CD45RA^int^ and display an antigen-experienced phenotype (CD27^−^).

Flow cytometry analysis of digested fresh tissue samples confirmed the accumulation of live (LIVEDEAD^®^-negative) B cells (CD45^+^CD19^+^HLA-DR^+^) in the inflammatory adventitial layer of the middle of the aneurysm, as compared to the neck (the portion of the aneurysm that is contiguous with the normal aorta). Local activation of B cells was suspected because most B cells had a memory-like phenotype (CD27^+^IgD^−^) and some harbored a GC-like phenotype (CD95^+^CD24^−^IgD^−^CD27^high^) (Figure 4). In addition, flow cytometry analysis of adventitial tissue samples revealed an increase in CD4^+^ T cells in the core of the aneurysm, most of which had a memory phenotype (CD45RA^−^). Interestingly, T follicular helper (Tfh) cells (PD1^+^CXCR5^+^Bcl6^high^) were present in the aortic wall as well and were antigen-experienced (CD27^−^) (Figure 4).

## Discussion

Takayasu arteritis is a rare form of chronic large vessel vasculitis of unknown origin involving the aorta and its major branches. Over the last decades, T cell-mediated immunity and inflammation have been implicated in the pathogenesis of this disease. Our data show that ectopic lymphoid neogenesis takes place in the aortic wall of patients with active TA and highlight the role of B cells in TA.

Using immunohistochemical examination, mature TLOs were detected in the adventitia of all but one TA aortic specimen. The accumulation of B cells within the adventitia, as well as their organization in TLOs suggests that B cells infiltrate the aortic wall in TA. The structural similarities between TLOs and B cells follicles found in secondary lymphoid organs suggest a local recruitment of naive cells *via* HEV (PNAd^+^), their activation, as well as the establishment of an immunological humoral memory supported by Tfh cells. We detected by immunostaining both TLOs and granulomas, each displaying distinct cellular composition and occupying different vascular niches. This suggests different functions and involvement in the activity of the disease with TLOs enhancing the destructive properties of granulomas and vice versa.

Analysis of immune adventitial cells revealed a high percentage of memory and antigen-experienced CD4^+^ T cells and also the presence of cells expressing canonical Tfh cell markers, such as CXCR5, Bcl6, and PD-1. These cells orchestrate B-cell activation, proliferation, and function (15). TLOs development and maintenance in TA may thus depend on the Tfh cell compartment as recently reported in atherosclerosis-prone mice (5). In addition, patients with TA are known to have enhanced interleukine-6 (IL-6) serum levels that parallel disease activity (16) and inhibition of IL-6 by the monoclonal anti-IL-6 receptor antibody tocilizumab is clearly efficient in TA (17). Interestingly, IL-6 is essential for B and Tfh cell differentiation (18) suggesting that immunotherapy against IL-6 could have dampened TLO development in the inflamed arteries of TA patients. Thus, our data suggest that the TLOs in TA can support antigen-driven clonal expansion and diversification and contain key elements for driving an immune pathogenic response that could last for decades if long lived plasma cells and memory CD4^+^ T cells are generated.

Tertiary lymphoid organs can develop in inflamed tissues with a frequency that varies greatly depending on the anatomical sites and diseases. Ectopic lymphoid tissue and lymphoid neogenesis have been observed in infection or immune disorders, including synovia in rheumatoid arthritis (19), salivary glands of patients with Sjogren’s syndrome (20), multiple sclerosis, inflammatory bowel diseases, and allografts (4). Although we still do not know what antigenic stimuli trigger their formation, TLOs cannot be considered as simple passive bystander markers of tissue inflammation because they are able to promote auto- or allo-antibody production and activating cellular effectors resulting in organ damage such as chronic inflammatory disorders and allograft rejection (3, 4).

In conclusion, TLOs are detected in the aortic adventitia of TA and comprise Tfh cells, clearly implicating B-cells in active TA. Deciphering whether TLOs are functional and allow the maturation of B cells and the production of antibodies remain to be formally demonstrated. In addition, understanding the respective involvement of local immunological reactions associated with TLO and granuloma formation in the pathogenesis of TA, as well as deciphering the cell types involved and the distinctive factors triggering the formation of each type of leukocyte aggregates, will lead to the development of new therapeutic approaches to treat patients with TA.

## Author Contributions

KS had full access to all of the data in the study and takes responsibility for the integrity of the data and the accuracy of the data analysis. Study design: MC, AN, and KS. Acquisition of data: MC, AG, PB, MM, FH, KB, NP, LD, QP, TP, AN, and KS. Analysis and interpretation of data: MC, AG, PB, FH, NP, LD, and KS. Manuscript preparation: MC, FH, AN, TP, and KS.

## Conflict of Interest Statement

The authors declare that the research was conducted in the absence of any commercial or financial relationships that could be construed as a potential conflict of interest.
